# Effects of C-reactive Protein and Homocysteine on Cytokine Production: Modulation by Pravastatin

**DOI:** 10.1111/j.1753-5174.2007.00003.x

**Published:** 2008-07

**Authors:** Yu Asanuma, Annette Oeser, Eran Stanley, David G Bailey, Ayumi Shintani, C Michael Stein

**Affiliations:** * Division of Rheumatology and Applied Immunology, Saitama Medical UniversitySaitama, Japan; † Division of Clinical Pharmacology, Vanderbilt University School of MedicineNashville, TN, USA; ‡ Division of General Internal Medicine, Vanderbilt University School of MedicineNashville, TN, USA; § Department of Medicine, Lawson Health Research Institute Health Sciences Centre LondonOntario, Canada

**Keywords:** Pravastatin, C-reactive protein, Interleukin-6

## Abstract

**Objective:**

C-reactive protein (CRP) and homocysteine are markers of cardiovascular risk that may have inflammatory effects. HMG coenzyme A reductase inhibitors (statins) have anti-inflammatory effects *in vitro,* but it is not clear if such responses *in vivo* are secondary to lipid lowering. We examined the hypothesis that CRP and homocysteine would stimulate cytokine release in human whole blood and that short-term treatment with a statin would inhibit it.

**Methods:**

The time course of IL-6 and MCP-1 production was determined in whole blood incubated with saline, 1 µg/mL lipopolysaccaride (LPS), 50 and 100 µM/L DL-homocysteine, and 5 µg/mL human recombinant CRP for 24 hours at 37°C under 5% CO_2_ atmosphere. Cytokine responses were determined in blood drawn from 15 healthy volunteers before and after administration of pravastatin 40 mg daily for 2 days.

**Results:**

Both human recombinant CRP and LPS significantly increased the production of IL-6 and MCP-1 in whole blood samples more than 4-fold (*P* < 0.001) but homocysteine did not. Oral administration of pravastatin, 40mg daily for 2 days, decreased CRP-stimulated IL-6 production by approximately 20% (*P* = 0.02) 6 hours after incubation, but did not affect MCP-1 production (*P* = 0.69). Pravastatin treatment did not affect LPS-stimulated MCP-1 but increased IL-6 modestly.

**Conclusions:**

CRP stimulated the production of the proatherogenic mediators MCP-1 and IL-6 in human whole blood, but homocysteine did not. CRP-stimulated production of IL-6, but not MCP-1, was modestly attenuated by short-term treatment with pravastatin.

Many studies have shown that C-reactive protein (CRP) and homocysteine concentrations are associated with increased cardiovascular risk [1,2]. Elevated concentrations of CRP are associated with increased risk of coronary events and mortality in patients with coronary artery disease, other high-risk individuals, and apparently healthy people, independent of other cardiovascular risk factors [3,4]. Similarly, increased homocysteine concentrations are an important risk factor for coronary heart disease in both healthy subjects [5] and patients with ischemic heart disease [6]. It is not clear to what extent homocysteine and CRP may contribute directly to coronary risk, but recent *in vitro* studies indicate that they may have pro-atherogenic effects.

Homocysteine induced the expression and secretion of the pro-atherogenic chemokine, monocyte chemoattractant protein-1 (MCP-1), and the cytokine, interleukin-8 (IL-8), in human aortic endothelial cells [7]. Similarly, although CRP has traditionally been considered to be a non-specific acute phase reactant, it induced monocyte activation and adhesion, and the expression and release of adhesion molecules, MCP-1 and IL-6 in human endothelial cells [8]. Thus, homocysteine and CRP appear to act not only as markers of cardiovascular risk, but based on *in vitro* studies, to induce inflammatory responses and thus perhaps play a direct role in the pathogenesis of atherosclerosis. However, whether these pro-inflammatory effects of CRP and homocysteine occur *in vivo* is not clear.

Drugs that inhibit hydroxymethyl glutaryl (HMG) coenzyme A reductase activity (statins) decrease the production of low-density lipoprotein (LDL) cholesterol and up-regulate the expression of LDL-receptors [9]. Statins reduce cardiovascular mortality, but appear to do so to a greater extent than would be predicted from the LDL cholesterol reduction achieved [10]. Accordingly, other mechanisms have been sought through which statins may decrease cardiovascular risk. Anti-oxidant, antiproliferative, immunomodulatory and anti-inflammatory effects have been identified *in vitro* and as a result the effects of statins have been described as pleiotropic [11]. However, little is known about the *in vivo* anti-inflammatory effects of statins and whether these are independent of cholesterol lowering.

Treatment with statins consistently decreases plasma CRP concentrations [12,13]. Statins also reduce the serum levels and the expression of proinflammatory cytokines and chemokines in leukocytes from treated hypercholesterolemic patients [14]. However, the effects of statins on CRP and cytokines *in vivo* have been studied after several weeks of treatment when effects on cholesterol have already occurred, and thus anti-inflammatory effects may be secondary to lower cholesterol concentrations. We postulated that if statins have a direct anti-inflammatory effect *in vivo* this effect would occur rapidly, as it does *in vitro*.

First, we examined the hypothesis that CRP and homocysteine would stimulate the production of IL-6 and MCP-1, proatherogenic cytokines, in whole blood and second, that this effect would be attenuated by short-term treatment with a statin.

## Methods

The study was approved by the Institutional Review Board of Vanderbilt University Hospital and all subjects gave written informed consent.

We studied 15 healthy subjects (6 male and 9 female; 6 Caucasian, 5 African American, 3 Asian and 1 Hispanic) who had not taken any medications for at least 7 days and no alcohol or caffeine for at least 2 days before the study. The subjects had an average (±SEM) age of 27 ± 1 years and weight of 73.1 ± 3.6 kg. Subjects were fasted overnight and venous blood (50 mL) was drawn. Subjects then swallowed pravastatin 40 mg with water in the presence of an investigator. Twenty four hours later subjects received a second 40 mg dose, again in the presence of an investigator, and 1.5 hours later, a time when peak concentrations of pravastatin are expected to occur, 50 mL blood was drawn. Several studies have shown that the mean time to reach maximum concentration of pravastatin is approximately 1.5 hours (range, 0.5–4 hours) [15], thus we drew blood for the *ex vivo* studies 1.5 hours after administration of pravastatin.

Venous blood from these volunteers was collected into tubes containing heparin, maintained at 37°C, and studied within 60 minutes. Cytokine production *ex vivo* was measured as previously described by ourselves and others [16,17]. Eight hundred µl of heparinized whole blood was incubated with 200 µL saline (control), lipopolysaccaride (LPS, Escherichia coli O111:B4 from Sigma, St. Louis, MO) (final concentration 1 µg/mL, n = 15), human recombinant CRP (Calbiochem, San Diego, CA) (final concentration 5 µg/mL, n = 15) or DL-homocysteine (Sigma, St Louis, MO) (final concentration 50 µM/L, n = 8, and 100 µM/L, n = 6) for 24 hours at 37°C under 5% CO_2_ atmosphere. At 0, 6, 10 and 24 hours samples were centrifuged in an Eppendorf microcentrifuge at 1,500 × g for 10 minutes. Supernatants were stored at –70°C until IL-6 and MCP-1 concentrations were measured. The 6-hour time-point was of primary interest as regards the effects of pravastatin because this was the shortest incubation period that we had shown in pilot studies resulted in a robust stimulation of cytokines by CRP. The experiments to determine the effects of homocysteine on cytokine production were discontinued when it was became clear after studying 7 subjects that there was no effect.

### Measurement of Cytokine and Pravastatin Concentrations

IL-6 and MCP-1 concentrations were measured using enzyme linked immunosorbent assay kits (R&D systems, Minneapolis, MN). All samples were analyzed in duplicate and results averaged. In order to correct for spontaneous cytokine generation, the net production of cytokines was calculated by subtracting the concentrations in the saline controls from those induced by LPS or CRP.

Pravastatin concentrations were measured by HPLC. A 100 mg C_18_ preparatory solid-phase extraction column (Sep-Pak Vac, Waters, Mississauga, ON) was initially washed with 1ml volumes of isopropyl alcohol, methanol and water. An aliquot (1,000 µL) of plasma was added and the column was washed three times with 1ml of acetic acid: methanol: water (2:10:88 v/v) and then three times with 1 mL ammonium hydroxide: methanol: water (2:10:88 v/v). The sample was eluted with 1ml methanol containing triethylamine (200 µL/100 mL) and it was evaporated to dryness at 40°C under a gentle stream of nitrogen. The residue was dissolved in an aliquot (100 µL) of HPLC mobile phase which consisted of acetonitrile: water (27:73 v/v) and triethylamine 500 µL/L at pH = 3.0 with phosphoric acid that also contained the internal standard, 5-methoxypsoralen, (Indofine Chemical Company Inc., Somerville, NJ) at a concentration of 500 ng/mL. The solution was filtered (0.45 µM) and a sample (30 µL) was injected onto a 150 mm × 3.2 mm Prodigy C_18_, 5 mm column (Phenomenex, Torrance, CA) at a mobile flow rate of 0.4 mL/min. Absorbance detection at 238 nm was used to monitor the effluent. The retention times of pravastatin and 5-methoxypsoralen were 13 and 23 minutes, respectively. The standard curve of pravastatin was linear over the range tested (0–200 ng/mL). The coefficient of variation was 3.3% at 25 ng/mL (n = 7) and the limit of detection was 5 ng/mL.

### Measurement of Endotoxin Concentration

Recombinant human CRP contains low concentrations of endotoxin which were measured using a limulus amebocyte lysate assay (Cape Cod, Falmouth, MA) which has a lower limit of detection of 0.005 Endotoxin Units (EU).

### Statistical Analysis

Results are expressed as mean ± SEM in the text and figures. Wilcoxon signed ranks tests were used to compare cytokine production in stimulated and in unstimulated whole blood and to compare stimulated IL-6 and MCP-1 production before and after the administration of pravastatin. Correlations were assessed by Spearman's rank order test. Values of *P* < 0.05 were considered significant. Statistical analysis was performed with SPSS 11.5 for Windows (SPSS, Chicago, IL).

## Results

### Effects of CRP and DL-homocysteine on IL-6 and MCP-1 Production

The major findings of this study are that human recombinant CRP, but not homocysteine, stimulates cytokine production in human whole blood and that short-term treatment with a statin modestly inhibits CRP-stimulated IL-6 but not MCP-1 production after 6 hours of incubation.

**Figure 1:**
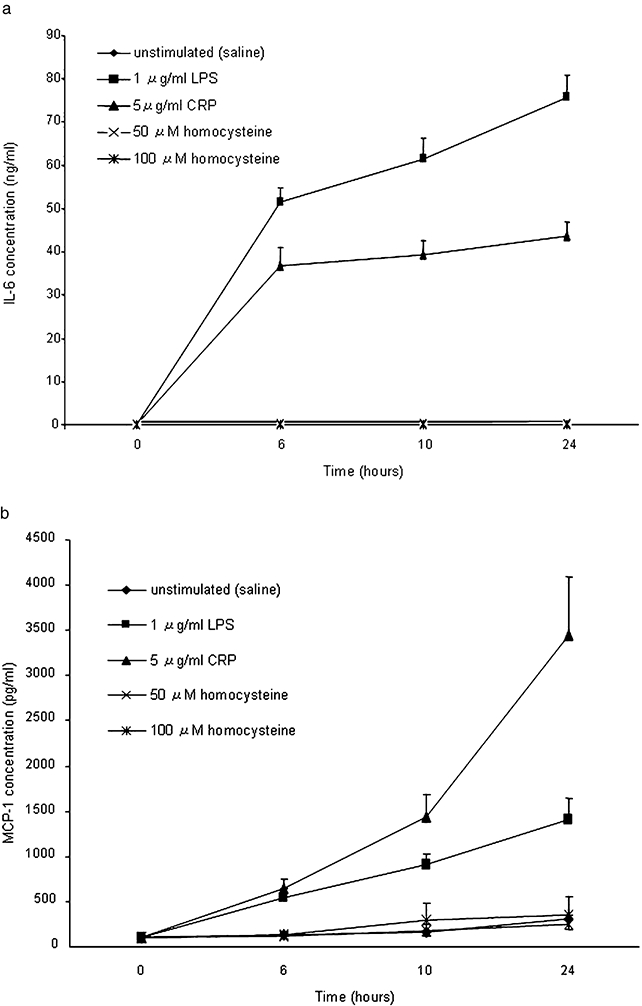
(a) Time course of IL-6 production in whole blood. (b) Time course of MCP-1 production in whole blood. LPS and recombinant CRP induced IL-6 and MCP-1 release in whole blood culture. Data are expressed as mean ± SEM. *P < 0.001 compared with control (IL-6 and MCP-1 production in unstimulated whole blood).

**Figure 2:**
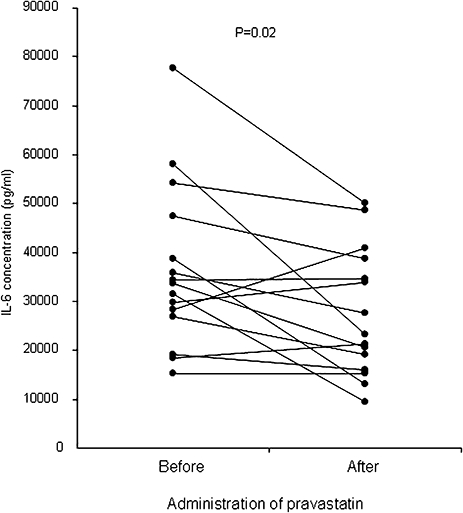
Effect of pravastatin on IL-6 production in whole blood during stimulation with 5 g/mL recombinant CRP for 6 hours (n = 15). Administration of pravastatin reduced CRP-stimulated IL-6 production by an average of –20% (*P*= 0.02).

LPS and recombinant CRP increased IL-6 (Figure 1a) and MCP-1 (Figure 1b) production significantly (all *P* values < 0.001 compared to saline control). This effect occurred rapidly, within 6 hours. Neither 50 nor 100 µM/L DL-homocysteine affected MCP-1 or IL-6 production significantly compared to saline (all *P* values > 0.07) (Figure 1a, b). Pravastatin reduced CRP-stimulated IL-6 production from 36.6 ± 4.3 ng/mL (median 34.1) to 27.4 ± 3.3 ng/mL (median 25.5), an average of –19.7% ± 8.3% after 6 hours of incubation (*P* = 0.02, Figure 2) but not after longer periods of incubation (Table 1). Pravastatin did not inhibit IL-6 production after 6 hours of incubation with LPS (55.1 ± 2.9 ng/mL compared to 51.4 ± 3.4 ng/mL before pravastatin, *P* = 0.09). IL-6 concentrations in LPS-stimulated whole blood for 10 hours tended to increase after subjects took pravastatin (*P* = 0.04, Table 1). Pravastatin did not affect CRP or LPS stimulated MCP-1 cytokine production (Table 2).

**Table 2:** MCP-1 production in recombinant CRP or LPS-stimulated whole blood before and after administration of pravastatin

	MCP-1 concentration (pg/ml)		
Incubation time	Before pravastatin	After pravastatin	*P*-values
Unstimulated (Saline)			
6 hours	105.2 ± 20.9	111.4 ± 21.3	0.53
10 hours	157.1 ± 35.0	150.6 ± 31.2	0.65
24 hours	303.6 ± 108.1	407.4 ± 450.2	0.43
5 µg/mL CRP			
6 hours	442.3 ± 85.6	417.4 ± 72.7	0.69
10 hours	1,291.0 ± 229.4	1,458.9 ± 227.6	0.31
24 hours	3,157.5 ± 632.3	3,083.0 ± 629.1	0.65
1 µg/mL LPS			
6 hours	530.3 ± 101.6	502.4 ± 97.0	0.87
10 hours	759.0 ± 441.6	750.7 ± 471.1	0.73
24 hours	1,139.0 ± 879.4	899.4 ± 884.1	0.10

Values are mean SEM from 15 experiments performed in duplicates.

The net production of MCP-1 was calculated by subtracting the concentration without stimulus from those induced by LPS or recombinant CRP.

Wilcoxon signed rank test was applied for statistical analysis.

**Table 1:** IL-6 production in recombinant CRP or LPS-stimulated whole blood before and after administration of pravastatin

	IL-6 concentration (ng/ml)		
Incubation time	Before pravastatin	After pravastatin	*P* values
Unstimulated (Saline)			
6 hours	0.09 ± 0.05	0.10 ± 0.05	0.83
10 hours	0.09 ± 0.05	0.13 ± 0.06	0.50
24 hours	0.13 ± 0.06	0.16 ± 0.06	0.78
5 µg/ml CRP			
6 hours	35.2 ± 17.5	25.5 ± 13.6	0.02
10 hours	39.3 ± 3.27	33.6 ± 3.90	0.14
24 hours	42.3 ± 3.28	40.6 ± 4.26	0.61
1 µg/ml LPS			
6 hours	51.4 ± 3.4	55.1 ± 2.9	0.09
10 hours	61.5 ± 4.69	69.9 ± 4.08	0.04
24 hours	75.9 ± 20.1	80.7 ± 18.8	0.36

Values are mean SEM from 15 experiments performed in duplicates.

The net production of IL-6 was calculated by subtracting the concentration without stimulus from those induced by LPS or recombinant CRP.

Wilcoxon signed rank test was applied for statistical analysis.

### Plasma Pravastatin Concentrations

The mean plasma pravastatin concentration was 50.3 ± 9.7 ng/mL (range 5–159.6 ng/mL). There was no significant correlation between plasma pravastatin concentrations and percent change in CRP-stimulated IL-6 (*r* = –0.31, *P* = 0.27) or MCP-1 concentrations (*r* = 0.16, *P* = 0.56), or LPS-stimulated IL-6 (*r* = –0.49, *P* = 0.07) and MCP-1 concentrations (*r* = –0.15, *P* = 0.27). There was a weak negative correlation between pravastatin concentration and absolute reduction in IL-6 concentration (*r* = –2.329, *P* = 0.02).

### Endotoxin Concentration

The average concentration of endotoxin in 5 µg/mL recombinant CRP (n = 4) was 0.38 ± 0.21 EU/mL and was >8 EU/mL in LPS 1 µg/mL, 100 ng/mL and 10 ng/mL, and 1.36 EU/mL in 1 ng/mL (n = 2, each).

## Discussion

We found that human recombinant CRP stimulated production of the inflammatory cytokines IL-6 and MCP-1 in whole blood but that homocysteine did not. Short-term treatment with an HMG co-enzyme A reductase inhibitor, pravastatin, reduced IL-6 production modestly after stimulation by CRP and tended to increase IL-6 production after LPS.

Increasing evidence implicates CRP as a contributor to the pathogenesis of atherosclerosis. CRP accumulates in the macrophage rich areas of the atherosclerotic plaque [18] and increases the expression and release of macrophage derived pro-inflammatory cytokines such as interleukin-1β and IL-6 [19,20]. In endothelial cells, CRP increased the expression of vascular cell adhesion molecule (VCAM-1) and intracellular adhesion molecule (ICAM-1), and increased MCP-1 production and tissue factor release [8,21]. Based on *in vitro* and *in vivo* data we selected the concentration of CRP for study, 5 µg/mL. Thus, in patients with stable or unstable angina, serum concentrations of CRP >3 µg/mL are associated with an increased risk of coronary events [22,23] and in vitro concentrations of 5 µg/mL had significant inflammatory effects [24].

We found that recombinant CRP markedly increased the production of IL-6 and MCP-1, cytokines that are thought to be important in the pathogenesis of atherosclerosis [25,26]. Human recombinant CRP is known to contain low concentrations of endotoxin. The question arises whether this could account for the ability of CRP to stimulate cytokine production. Various strategies, such as boiling CRP to destroy it and thus isolate the contribution of endotoxin to observed responses, or passing CRP through columns containing polymyxin to remove endotoxin, have been explored. However, low levels of endotoxin are present in apparently healthy subjects [27,28] and have been associated with risk of atherosclerosis [28]. Much higher levels of endotoxin (>50 pg/mL) are observed in subjects with chronic or recurrent bacterial infections, another risk factor for atherosclerosis [29]. Thus, considering the possibility that CRP and endotoxin are both risk factors for atherosclerosis and may act synergistically *in vitro*[20] and *in vivo*, our strategy was not to remove the endotoxin present in CRP but to measure the concentrations present. The concentrations of endotoxin present in CRP were low, generally less than 0.5 EU/mL, are unlikely to account for the cytokine stimulation by CRP [30]. Thus our studies suggest that human recombinant CRP in the presence of very low levels of endotoxin stimulates IL-6 and MCP-1 production in whole blood.

Treatment with a statin for several weeks has been shown to decrease CRP production [12] and to reduce the expression of IL-6, IL-8, and MCP-1 mRNA in peripheral blood cells and to decrease serum cytokine concentrations [31,32]. Statins have rapid anti-inflammatory effects *in vitro*decreasing the production of cytokines within hours [8,33,34]. Studies that have examined the effects of statins on cytokine production *ex vivo* have done so after several weeks of treatment [12] and thus have not separated out direct antiinflammatory effects from those due to the reduction in lipids and CRP [31]. Treatment with a statin decreases CRP and LDL cholesterol concentrations rapidly, with effects seen after 14 days [35]. However, treatment for 3 to 6 days does not alter LDL cholesterol concentrations [36] indicating that the effects noted in the present study after two doses are not likely to be secondary to lowering of cholesterol.

We chose to study pravastatin; there were several rationales for this choice. First, it is widely used clinically, and since it is not a CYP3A4 substrate has fewer interactions with other drugs. Second, an anti-inflammatory effect of pravastatin has been well demonstrated. Randomized, double-blind studies have shown that several statins decrease concentrations of C-reactive protein (CRP) and that the reductions obtained with different statins were similar [12]. However, the effect of pravastatin on CRP was independent of its lipid-lowering effects [13].

The finding that treatment with a statin has a rapid inhibitory effect on CRP stimulated IL-6 production is of interest, not only because it demonstrates a rapid anti-inflammatory action of a statin *ex vivo,*but also because IL-6 is thought to be one of the prime regulators of CRP production [37]. Furthermore, our findings suggest that CRP may stimulate IL-6. Concordant with this notion is the observation that CRP and IL-6 concentrations are correlated [38,39]. Thus the inhibitory effect of statins on CRP could be mediated in part by effects on IL-6.

The effects of pravastatin on CRP stimulated IL-6 production varied among individuals. Since pravastatin concentrations are known to vary more than 10-fold among individuals receiving the same dose [40], we examined the possible relationship between pravastatin concentrations and the change in cytokine production. There was no correlation between pravastatin concentrations and effect suggesting that variability in drug concentrations does not contribute substantially to the differences observed among individuals.

An inhibitory effect of pravastatin was observed on CRP-stimulated IL-6 after 6-hour incubation but not after longer incubation times. It is likely that multiple pathways of IL-6 activation are secondarily activated by the CRP-stimulated inflammation and that these may not be sensitive to pravastatin. Pravastatin did not affect either MCP-1 production and tended to increase LPS-stimulated IL-6 responses. The explanation for this is not clear but it suggests that the anti-inflammatory effects of statins may be specific to particular cytokines and stimuli.

The lack of effect of homocysteine on cytokine production was unexpected. Several lines of evidence suggest that hyperhomocysteinemia may be more than a marker of cardiovascular risk. Homocysteine is rapidly auto-oxidized resulting in the production of potent reactive oxygen species that result in damage to endothelial cells [41]. Homocysteine also limits the bioavailability of nitric oxide, and in cultured human aortic endothelial cells and THP-1 monocytes, decreases the expression and secretion of MCP-1 [7,38]. However, the effects of homocysteine on isolated cells in culture may not be representative of a more physiological milieu. An advantage of the *ex vivo* whole blood cytokine stimulation technique is that it provides a composite measure of response that can involve several different cell types activated in a biologically relevant environment.

Using concentrations of homocysteine that had inflammatory effects *in vitro,* and are in the range that may occur in patients with hyperhomocysteinemia [41], we found no effect on IL-6 or MCP-1 production in whole blood over 24 hours of incubation. This contrasts with responses in human aortic endothelial cells where responses to homocysteine occurred rapidly, plateauing within 2–8 hours, and were inhibited by cycloheximide indicating that *de novo* protein synthesis had occurred [7]. A possible explanation for the different findings may be that the whole blood environment provides antioxidant defenses that are not present in cell culture and may thus attenuate the pro-inflammatory effects of homocysteine that are thought to be mediated by free radicals [42]. Thus, our findings suggest that the previous observations that homocysteine stimulated the production of cytokines in isolated cells *in vitro* may not be generalizable to the *in vivo* situation.

A limitation of this study is that we did not investigate the effect of escalating doses of pravastatin. Several randomized trials have shown that pravastatin 40 mg daily reduced CRP concentrations significantly [12,13,43], but there is no dose-response information for this response. Therefore, we chose a dose of pravastatin 40 mg daily as most likely to show an effect. Future studies could define the dose-response more completely.

In summary, human recombinant CRP with low concentrations of endotoxin stimulated the production of the proatherogenic mediators MCP-1 and IL-6 in human whole blood, but homocysteine did not. CRP-stimulated production of IL-6, but not MCP-1, was modestly attenuated by short-term treatment with pravastatin indicating that treatment with a statin may have rapidly anti-inflammatory actions *in vivo* and that these effects may be specific to particular cytokines and stimuli.
